# The relationship between emotional eating, mindful eating, and depression in young adults

**DOI:** 10.1002/fsn3.4028

**Published:** 2024-02-18

**Authors:** Ziya Erokay Metin, Nursena Bayrak, Özge Mengi Çelik, Muzaffer Akkoca

**Affiliations:** ^1^ Department of Nutrition and Dietetics, Gülhane Health Sciences Faculty University of Health Sciences Ankara Turkey; ^2^ Department of General Surgery University of Health Sciences Etlik City Hospital Ankara Turkey

**Keywords:** depression, eating behavior, emotional eating, mindful eating

## Abstract

The aim of this study was to examine the relationship between eating behavior, eating awareness, and depression in young adults. Young adults aged between 18 and 25 years were included in the study. Questionnaires and body composition measurements were made face‐to‐face by the researcher in the interview room. The demographic characteristics were questioned through the questionnaire form. Three Factor Eating Questionnaire (TFEQ), Mindful Eating Questionnaire (MEQ), and Beck Depression Inventory (BDI) were used. It was determined that the Mindful Eating Questionnaire total score and Beck Depression Inventory total score affected emotional eating. A statistically significant positive correlation was found between the Beck Depression Inventory total score and the Three Factor Eating Questionnaire sub‐scores. There was a statistically significant negative correlation Beck Depression Inventory total score and the Mindful Eating Questionnaire total score and sub‐scores of mindless eating and emotional eating. In summary, depression seems to be a factor that triggers emotional eating, and emotional eating is a factor that negatively affects mindful eating.

## Graphical abstract

Depression affects emotional eating. Emotional eating affects mindful eating.

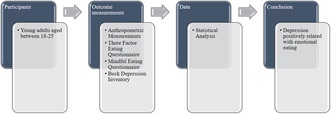

## 1 INTRODUCTION

Overweight and obesity are becoming increasingly common, posing a severe risk to the public's health (Donofry et al., 2020). Obesity is linked to systemic conditions such as metabolic syndrome, diabetes, hypertension, and cardiovascular disorders. In addition to these conditions, there is interest in the connection between depression and obesity (Stunkard et al., 2003). Obesity and mental disorders such as anxiety and depression have been linked to studies in both directions (Rajan & Menon, 2017; Vittengl, 2018).

Obesity and depression may be related; however, the exact nature of this connection is unclear (APA, 2013). Eating behavior disorders are commonly observed in both obesity and depression. Increasing food consumption to alleviate or eliminate negative emotions may explain the link between gaining weight and depression (Faith et al., 2011; Gibson, 2012). In a study examining the relationship between eating behavior and depression, it was reported that emotional eating was more common in obese adults with depression symptoms than in those who did not (Goldschmidt et al., 2014). Emotional eating and depression have been demonstrated to be substantially correlated in another study looking at the association among emotional eating and depression in overweight and obese people (Masheb & Grilo, 2006). On the other hand, dieting is also defined as a risk factor for the formation of depression and negative eating behaviors (Goldschmidt et al., 2016; Hilbert et al., 2014; Johnson & Wardle, 2005; Stice et al., 2000). Diet restriction theory, which is one of the theories that draw attention to this relationship, argues that overeating occurs with increased dependence on external cues instead of internal hunger cues (Herman & Mack, 1975). In this context, Konttinen et al.'s study on adults revealed that emotional eating and depressed symptoms have an impact on harmful food choices. Additionally, the same study's authors claimed that emotional eating can account for the association between consuming sweets and depression (Konttinen et al., 2010).

Studies to reduce the symptoms of depression, which is a common and negative health condition with a high burden of disease, have started to be carried out recently focusing on mindful interventions (Hofmann et al., 2010; Keng et al., 2011). Raising mindfulness has been reported to reduce depression levels, and individuals with high mindful eating scores have been reported to have lower levels of depression (Winkens et al., 2018). Winkens et al. reported a negative correlation between depressive symptoms and two of the mindful eating domains focused on eating and eating with awareness (Winkens et al., 2020). Another study showed that binge eating behavior and mood are linked to mindful eating (Giannopoulou et al., 2020).

The purpose of this study was to investigate the link between young adults' eating behaviors, mindful eating, and depression. The main questions are;

Q1: Is there a relationship between the three‐factor eating questionnaire (TFEQ) sub‐factor scores and the Beck depression inventory (BDI) scores?
Q2: Is there a relationship between emotional eating sub‐scores and the mindful eating questionnaire (MEQ) total scores?
Q3: Does depression affect emotional eating?

## 2 METHODS

Young adults aged between 18 and 25 years were included in the study. Of the subjects who participated in the study, 119 were male (57.2%) and 89 were female (42.8%). After the individuals read and signed the voluntary consent form, they were evaluated by the researchers according to the inclusion and exclusion criteria.

Inclusion criteria:

1. To be between the ages of 18 and 25,

Exclusion criteria:

1. Pregnant and lactating women,
2. People with any chronic disease,
3. People with any syndrome and/or systemic disease,
4. Those who take medications (cortisone, antidepressants, metformin, etc.) that will affect their appetite,
5. Users of hormone supplements,
6. People on an energy‐restricted diet.

The eligible volunteers who were evaluated according to the inclusion and exclusion criteria were included in the study. Individuals were called for questionnaires and measurements by appointment. Questionnaires and body composition measurements were made face‐to‐face by the researcher in the interview room. The demographic characteristics were questioned through the questionnaire form. Three Factor Eating Questionnaire (TFEQ), Mindful Eating Questionnaire (MEQ), and Beck Depression Inventory (BDI) were used. Before starting the study, University of Health Sciences Gülhane Scientific Research Ethics Committee approved the study protocol. All procedures in the study were carried out in accordance with the Declaration of Helsinki.

### 2.1 Anthropometric measurements and body composition

Anthropometric measurements (body weight and height) were taken in accordance with the technique. The body mass index (BMI) value was calculated by dividing the body weight by the square of the height (James, 1998). Body composition measurements were performed with the TANITA BC 418 ST body composition measuring device. The measurement of this device is based on the bioelectrical impedance analysis (BIA) method (Pietrobelli et al., 2004).

### 2.2 Three factor eating questionnaire (TFEQ)

The scale with 21 questions, which was conducted by Karakuş et al. for validity and reliability studies for Turkish society, was used. It is in the quadruple Likert format, which consists of cognitive restriction, uncontrolled eating, and emotional eating sub‐factors, and where the responses are absolutely wrong, mostly wrong, mostly true, absolutely true. Uncontrolled eating refers to loss of control while eating due to hunger or any external stimulus. The lowest score that can be obtained from this sub‐factor is 9, and the highest score is 36. The deliberate limiting of food consumption to manage body weight and shape is referred to as cognitive restriction. The lowest score that can be obtained for this sub‐factor is 6, and the highest score is 24. Emotional eating examines overeating in negative emotional situations (anger, sadness, stress, etc.). The lowest score that can be obtained from this sub‐factor is 6, and the highest score is 24. A high score from any sub‐factor of the scale indicates that the eating behavior related to that factor is high (Karakuş et al., 2016).

### 2.3 Mindful eating questionnaire (MEQ)

The scale was developed by Baer et al. (2006) in 2006 in order to measure the quality of attention paid to eating. The scale, a validity and reliability study conducted by Köse et al. (2016) in 2016, consists of 30 questions. In the application of the scale, a 5‐point Likert scale (1: never, 2: rarely, 3: sometimes, 4: often, 5: always) is used. The scale consists of seven sub‐factors: disinhibition, emotional eating, eating control, mindfulness, eating discipline, conscious eating, and interference. High scores are interpreted positively for total score and all sub‐factors.

### 2.4 Beck depression inventory (BDI)

The Beck Depression Inventory, developed by Beck and his colleagues (Beck et al., 1987), was used to determine the presence and degree of depression. Its validity and reliability study was conducted by Aktürk et al. in (2005). The BDI consists of a total of 21 items, each of which is scored between 0 and 3. The minimum‐maximum score range that can be obtained from the BDI is 0–63. A high score on the scale indicates that the severity or level of depression is high. The cut‐off score of the scale was determined to be 17, and it was reported that a score of 17 and above can obtain the degree of differentiation of depression requiring treatment with 90% accuracy (Aktürk et al., 2005).

### 2.5 Statistical analysis

All analyses were performed using the Statistical Package for the Social Sciences (version 22.0) program. Descriptive statistics including mean, standard deviation, number, and percentage were used to evaluate the data. The Pearson correlation coefficient is used to show the correlations between the variables. Regression analysis was performed for the prediction of eating behaviors and mindful eating. The results were evaluated at the 95% confidence interval, *p* < .05, and *p* < .001 significance level.

## 3 RESULTS

The general characteristics of individuals are given in Table 1. The mean age of the individuals was 21.2 ± 1.67 years, and the mean BMI was 22.6 ± 3.29 kg/m^2^. The mean number of main meals of the individuals was 2.6 ± 0.59, and the mean number of snacks was 1.3 ± 1.04. The mean Mindful Eating Questionnaire total score was 3.1 ± 0.37, and the mean Beck Depression Inventory total score was 31.9 ± 9.10.

**TABLE 1:** General characteristics of individuals.

Variables	$\bar{X}$ ± SD
Age (years)	21.2 ± 1.67
Gender	
Female	89 (42.8%)
Male	119 (57.2%)
BMI (kg/m^2^)	22.6 ± 3.29
Body fat (%)	
Female	25.8 ± 5.95
Male	14.2 ± 5.32
Number of main meals	2.6 ± 0.59
Number of snacks	1.3 ± 1.04
The Three Factor Eating Questionnaire (TFEQ)	
Cognitive restriction	29.6 ± 21.42
Emotional eating	27.1 ± 23.93
Uncontrolled eating	43.2 ± 21.24
The Mindful Eating Questionnaire (MEQ)	
Total score	3.1 ± 0.37
Disinhibition (mindless eating)	3.2 ± 0.80
Emotional eating	3.5 ± 0.80
Eating control	2.9 ± 0.52
Mindfulness	3.0 ± 0.51
Eating discipline	2.9 ± 0.76
Conscious nutrition	3.0 ± 0.54
Interference	3.2 ± 0.93
Beck Depression Inventory total score	31.9 ± 9.10

Abbreviation: BMI, body mass index.

The relationship between the Mindful Eating Questionnaire, Three Factor Eating Questionnaire, and Beck Depression Inventory was given in Table 2. A statistically significant positive correlation was found between Beck Depression Inventory total score and the Three Factor Eating Questionnaire sub‐scores. There was a statistically significant negative correlation Beck Depression Inventory total score and the Mindful Eating Questionnaire total score and sub‐scores of mindless eating and emotional eating. A statistically significant positive correlation was found between the cognitive restriction score and the Mindful Eating Questionnaire total score. Also, there was a statistically significant negative correlation between the Mindful Eating Questionnaire total score and scores of emotional eating and uncontrolled eating (*p* < .05).

**TABLE 2:** The relationship between the Mindful Eating Questionnaire, the Three Factor Eating Questionnaire, and the Beck Depression Inventory.

	The Three Factor Eating Questionnaire			The Mindful Eating Questionnaire								Beck Depression Inventory
	Cognitive restriction	Emotional eating	Uncontrolled eating	Total score	Mindless eating	Emotional eating	Eating control	Mindfulness	Eating discipline	Conscious nutrition	Interference	Total score
The Three Factor Eating Questionnaire												
Cognitive restriction	–	0.251**	0.004	0.165*	0.069	−0.029	0.048	−0.178*	0.298**	0.222**	0.298**	0.201**
Emotional eating		–	0.368**	−0.344**	−0.230**	−0.577**	−0.120	−0.057	0.028	−0.083	−0.072	0.218**
Uncontrolled eating			–	−0.598**	−0.709**	−0.378**	−0.247**	−0.020	−0.136*	−0.320**	−0.313**	0.152*
The Mindful Eating Questionnaire												
Total score				–	0.747**	0.692**	0.501**	0.200**	0.490**	0.535**	0.586**	−0.144*
Mindless eating					–	0.502**	0.300**	−0.050	0.139*	0.284**	0.413**	−0.184**
Emotional eating						–	0.276**	−0.073	0.103	0.180**	0.350**	−0.156*
Eating control							–	0.096	0.078	0.224**	0.111	−0.049
Mindfulness								–	0.058	−0.054	−0.017	−0.028
Eating discipline									–	0.246**	0.309**	−0.059
Conscious nutrition										–	0.222**	0.067
Interference											–	0.058
Beck Depression Inventory												
Total score												–

* *p* < .05.

** *p* < .001.

When the factors that could affect the Mindful Eating Questionnaire total score was evaluated with linear regression analysis and the model was deemed important (*R* ^2^ = .424; *p* < .001). It was determined that scores of cognitive restriction, emotional eating and uncontrolled eating affected mindful eating (*p* < .05) (Table 3).

**TABLE 3:** Linear regression model for mindful eating prediction.

Model	The Mindful Eating Questionnaire total score		
	*β*	*t*	*p*‐Value
Beck Depression Inventory total score	−.069	−1.245	.214
Cognitive restriction score	.230	4.108	**<.001** *
Emotional eating score	−.197	−3.286	**.001** *
Uncontrolled eating score	−.516	−8.917	**<.001** *
		***R*** ^**2**^ **= .424; *p* < .001** *	

*Note*: Bold values show statistically significance.

* *p* < .05.

When the factors that could affect the TFEQ sub‐factors scores were evaluated with linear regression analysis, all models were deemed important (respectively *R* ^2^ = .368; *p* < .001, *R* ^2^ = .106; *p* < .001, *R* ^2^ = .152; *p* < .001). Mindful Eating Questionnaire total score was determined to affect uncontrolled eating (*p* < .05). It was determined that BMI, Mindful Eating Questionnaire total score, and Beck Depression Inventory total score affected cognitive restriction (*p* < .05). Also, it was determined that Mindful Eating Questionnaire total score and Beck Depression Inventory total score affected emotional eating (Table 4).

**TABLE 4:** Linear regression model for eating behaviors.

Model	TFEQ sub‐factors								
	Uncontrolled eating			Cognitive restriction			Emotional eating		
	*β*	*t*	*p*‐Value	*β*	*t*	*p*‐Value	*β*	*t*	*p*‐Value
BMI (kg/m^2^)	−.079	−1.414	.159	.166	2.482	**.014** *	−.065	−1.001	.318
Mindful Eating Questionnaire Total Score	−.594	−10.527	**<.001** *	.210	3.124	**<.002** *	−.324	−4.958	**<.001** *
Beck Depression Inventory total score	.076	1.337	.183	.212	3.149	**<.002** *	.179	2.728	**.007** *
		***R*** ^**2**^ **= .368; *p* < .001** *			***R*** ^**2**^ **= .106; *p* < .001** *			***R*** ^**2**^ **= .152; *p* < .001** *	

*Note*: Bold values show statistically significance.

* *p* < .05.

## 4 DISCUSSION

Depression is one of the important factors that can cause many diseases. It is of concern not only because it occurs in young populations during rapid transition periods but also because its prevalence has increased significantly in recent years (Thapar et al., 2022) and the literature indicates that lifestyle behaviors are associated with mental health (Berk et al., 2013). The relationship between eating behavior, which is one of the important lifestyle behaviors, and depression has recently attracted attention, and studies report a relationship between depression and eating behavior, although the underlying causes cannot be fully explained (Ljungberg et al., 2020). In addition, it is suggested that eating behavior and especially emotional eating may have an effect on mindful eating, which is demonstrated to be a key tool in the fight against obesity (Mantzios & Wilson, 2015). In this context, we conducted this study in which we examined the relationship among eating behavior, mindful eating, and depression in young adults, and according to the data we obtained, our main findings are as follows; (i) depression increases emotional eating, (ii) emotional eating decreases mindful eating, (iii) mindful eating decreases emotional eating and uncontrolled eating, while increasing cognitive restriction.

Depression may trigger food intake in individuals with emotional eating (Konttinen et al., 2010). Studies show that individuals with high depressive symptoms have more emotional eating (Ouwens et al., 2009; Paans et al., 2018). In addition, the results of some studies suggest that emotional eating is the bridge between depression and weight gain (Konttinen et al., 2019; van Strien, Konttinen, et al., 2016; Vittengl, 2018). The findings of the study by van Strien et al. support this situation. According to the authors, emotional eating may act as a mediator in Dutch moms' 5‐year weight gain and depression (van Strien, Winkens, et al., 2016). Similar to previous studies, we found a positive correlation between depression and emotional eating in our study. We also observed that BDI total scores affect emotional eating according to the regression model we created. The high emotional eating of individuals with high levels of depression may cause an increase in the energy intake of depressed individuals and, consequently, an increase in BMI (Konttinen et al., 2019). In addition, unlike previous studies, we investigated the relationship between emotional eating and mindful eating of young adults in this study. We discovered a substantial inverse relationship between emotional eating and mindful eating using our correlation analysis, and we found that emotional eating is a factor that has a negative impact on mindful eating using our regression analyses. In compliance with previous studies and our findings, it can be said that depression may increase emotional eating and emotional eating may decrease mindful eating. However, we did not find an effect of BDI total scores on MEQ total scores. On the other hand, according to our regression model, we also observed that mindful eating is a factor that negatively affects emotional eating. In this context, the relationship between mindful eating and emotional eating is supportive of prospective studies on mindful eating interventions and strategies to be developed for obesity.

The issue of mindfulness on the way to change eating behavior in a positive way attracts both research and clinical interest. However, intervention studies examining the effects of eating awareness on emotional eating are very limited (Katterman et al., 2014). The methodologies of these studies in the literature differ and report contradictory results (Kearney et al., 2012; Leahey et al., 2008; Timmerman & Brown, 2012). Lahey et al. initially included individuals with high levels of emotional eating in their study and showed that mindfulness intervention reduced emotional eating (Leahey et al., 2008). In contrast, studies that did not find a significant difference in emotional eating levels included individuals with low initial emotional eating levels (Kearney et al., 2012; Timmerman & Brown, 2012). A positive eating behavior change was also reported in a mindfulness intervention study on adult women with emotional eating (Beccia et al., 2020). We, on the other hand, concluded that although our study was of a cross‐sectional design, mindful eating is a factor that can affect eating behavior. According to our regression model, we observed that the eating behavior sub‐components of uncontrolled eating, cognitive restriction, and emotional eating, which we measured with the TFEQ, were affected by mindful eating scores. These results reveal that emotional eating should not be ignored in mindful eating interventions.

Our study has a number of strengths, such as evaluating the effect of depression on eating behaviors and mindful eating. Moreover, we evaluated the relationship between emotional eating and mindful eating. However, there were several limitations that may warrant attention in future research. Firstly, dietary records were not taken. By taking dietary records, diet quality and the relation between diet quality and depression can be evaluated. A second limitation was that the study design was cross‐sectional.

In summary, depression seems to be a factor that triggers emotional eating and emotional eating is a factor that negatively affects mindful eating. Although studies on mindful eating in the medical nutrition therapy of obesity are promising, factors such as depression and emotional eating should not be ignored. Although it is difficult to establish a direct link between depression and obesity, emotional eating can be considered a mediator. In this context, examining the effects of emotional eating and depression in randomized controlled studies to be designed for eating awareness practices will increase the effectiveness.

## AUTHOR CONTRIBUTIONS

**Ziya Erokay Metin:** Conceptualization (equal); data curation (equal); formal analysis (equal); investigation (equal); methodology (equal); supervision (equal); writing – original draft (equal); writing – review and editing (equal). **Nursena Bayrak:** Conceptualization (equal); data curation (equal); formal analysis (equal); investigation (equal); methodology (equal); writing – original draft (equal). **Özge Mengi Çelik:** Data curation (equal); formal analysis (equal); writing – original draft (equal); writing – review and editing (equal). **Muzaffer Akkoca:** Writing – review and editing (equal).

## CONFLICT OF INTEREST STATEMENT

The authors have declared no conflicts of interest.

## Data Availability

Data is available upon request from the authors.
